# A Systematic Review on the Correlations between Left Atrial Strain and Cardiovascular Outcomes in Chronic Kidney Disease Patients

**DOI:** 10.3390/diagnostics11040671

**Published:** 2021-04-08

**Authors:** Ana Tanasa, Alexandru Burlacu, Cristina Popa, Mehmet Kanbay, Crischentian Brinza, Liviu Macovei, Radu Crisan-Dabija, Adrian Covic

**Affiliations:** 1 Nephrology Clinic, Dialysis, and Renal Transplant Center—‘C.I. Parhon’ University Hospital, 700503 Iasi, Romania; tanasa.ana@umfiasi.ro (A.T.); cristina-adriana_cg_rusu@d.umfiasi.ro (C.P.); adrian.covic@umfiasi.ro (A.C.); 2 Department of Internal Medicine, University of Medicine and Pharmacy “Grigore T Popa”, 700115 Iasi, Romania; crischentian-branza@email.umfiasi.ro (C.B.); liviu.macovei@umfiasi.ro (L.M.); radu.dabija@umfiasi.ro (R.C.-D.); 3 Institute of Cardiovascular Diseases “Prof. Dr. George I.M. Georgescu”, 700503 Iasi, Romania; 4 Department of Medicine, Division of Nephrology, Koc University School of Medicine, 34450 Istanbul, Turkey; drkanbay@yahoo.com

**Keywords:** left atrial strain, chronic kidney disease, cardiovascular outcomes, systematic review, early marker

## Abstract

Left atrial strain (LASr) represents a relatively new but promising technique for left atrial and left ventricle function evaluation. LASr was strongly linked to myocardial fibrosis and endocardial thickness, suggesting the utility of LASr in subclinical cardiac dysfunction detection. As CKD negatively impacts cardiovascular risk and mortality, underlying structural and functional abnormalities of cardiac remodeling are widely investigated. LASr could be used in LV diastolic dysfunction grading with an excellent discriminatory power. Our objectives were to assess the impact and existing correlations between LASr and cardiovascular outcomes, as reported in clinical trials, including patients with CKD. We searched PubMed, Web of Science, Embase, and the Cochrane Central Register of Controlled Trials for full-text papers. As reported in clinical studies, LASr was associated with adverse cardiovascular outcomes, including cardiovascular death and major adverse cardiovascular events (HR 0.89, 95% CI, 0.84–0.93, *p* < 0.01), paroxysmal atrial fibrillation (OR 0.847, 95% CI, 0.760–0.944, *p* = 0.003), reduced exercise capacity (AUC 0.83, 95% CI, 0.78–0.88, *p* < 0.01), diastolic dysfunction (*p* < 0.05), and estimated pulmonary capillary wedge pressure (*p* < 0.001). Despite limitations attributed to LA deformation imaging (image quality, inter-observer variability, software necessity, learning curve), LASr constitutes a promising marker for cardiovascular events prediction and risk evaluation in patients with CKD.

## 1. Introduction

Chronic kidney disease (CKD) represents a significant health care problem with a global prevalence of 697.5 million cases and 1.2 million deaths in 2017 [1]. It is well-known that patients with CKD carry a high cardiovascular risk. On the one hand, CKD represents an independent cardiovascular risk factor and a coronary artery disease (CAD) equivalent for all-cause mortality; on the other hand, cardiovascular diseases (CVD) may contribute to further CKD progression to end-stage kidney disease (ESKD) [2,3,4,5,6,7]. This interdependence becomes more evident as CKD and CVD share similar risk factors (age, diabetes mellitus, dyslipidemia, hypertension, family history, smoking), thus amplifying the disease’s burden [8].

Moreover, studies suggest that mortality is primarily driven by CVD in patients with CKD, especially when the estimated glomerular filtration rate (eGFR) is under 60 mL/min/1.73 m^2^. Among CVD, ischemic heart disease and heart failure are the main factors associated with an increased CKD patients mortality [9]. Additionally, CKD is linked to a 2–3-fold higher rate of atrial fibrillation (AF), further increasing the risk of all-cause mortality by 23% and the risk of cardiovascular mortality by 45% [10].

As CKD negatively impacts cardiovascular risk and mortality, underlying structural and functional abnormalities of cardiac remodeling are widely investigated in the literature. Many physiopathological mechanisms are incriminated in CVD development: uremic toxins, left ventricular hypertrophy, myocardial fibrosis, inflammation, oxidative stress, growth factors, Klotho proteins, fibroblast growth factor 23, and the soluble receptor for advanced glycation end-products [11].

In the last years, great importance was given to the prevention of CVD in patients with CKD. A better stratification of cardiovascular and mortality risks represented one of the most important research directions, using dedicated clinical tools or novel biomarkers and imagistic techniques [12,13,14,15,16,17]. However, neither Framingham risk score nor pooled cohort equations from the American College of Cardiology/American Heart Association included markers of kidney function in the final prediction model, highlighting the need for an adapted and accurate tool for predicting adverse events in CKD [18,19].

Left atrial (LA) volume and dimensions evaluated by two-dimensional transthoracic echocardiography represent established imagistic markers associated with an increased risk of CVD, mortality, and AF in patients with CKD, including ESKD [20,21,22]. Once speckle tracking echocardiography became available, clinical studies revealed its superiority compared to traditional echocardiography in cardiac dysfunction detection by assessing ventricular and atrial myocardial deformation [23,24,25].

As new evidence has emerged in favor of deformation imaging, the European Association of Cardiovascular Imaging/American Society of Echocardiography/Industry Task Force published a consensus regarding standardization of left atrial, right ventricle, and right atrial deformation imaging in order to improve the quality and replicability of further research [26]. Briefly, the Task Force recommends to use an apical four-chamber view in order to determine LA strain (LASr), but a biplane method using in addition a two-chamber view can be also appropriate. Endocardial and epicardial borders are defined manually or automatically, so that only the wall of LA is included in the analysis and pericardium is excluded. From the available parameters, the Task Force recommends to use LA global strain (LAGS) and to avoid evaluation of radial or transverse strain.

LASr represents a relatively new but promising technique for LA and left ventricle (LV) function evaluation [27]. A recent study reported a stronger association of LASr with mean pulmonary arterial wedge pressure measured invasively (area under curve, AUC 0.80, *p* < 0.001) than in the case of standard echocardiographic measures [28]. Moreover, LASr was strongly linked to myocardial fibrosis (*p* < 0.0001) and endocardial thickness (*p* = 0.0001), suggesting the utility of LASr in subclinical cardiac dysfunction detection [29]. LASr could be used in LV diastolic dysfunction grading with an excellent discriminatory power (AUC 0.86–0.91) [30].

Our objectives were to assess the impact and existing correlations between LASr and cardiovascular outcomes, as reported in clinical trials, including patients with CKD.

## 2. Materials and Methods

We used Preferred Reporting Items for Systematic Review and Meta-Analysis (PRISMA) checklist in conducting the systematic review [31].

### 2.1. Data Sources

The search was performed in one step, from 12 January 2021, to 25 January 2021, in PubMed, Web of Science, Embase, and the Cochrane Central Register of Controlled Trials. The time of publication was restricted to the interval between January 2000 and January 2021. The following terms were used in searching process: “left atrium”, “strain”, “deformation imaging”, “chronic kidney disease”, “cardiovascular”, “outcomes”, “risk” and “mortality”. Additionally, the search was restricted to trials involving humans. The reference sections of relevant articles were also searched manually for additional publications. Two independent reviewers (A.T., C.B.) selected studies after screening the title and abstract.

### 2.2. Study Selection

Studies were considered for inclusion in the systematic review if they met several criteria, according to the PICOTS framework (population, intervention, comparator, outcome, time, setting): (1) at least ten patients aged > 18 years were included; (2) patients with eGFR < 60 mL/min/1.73 m^2^ were included; (3) original data were reported regarding LASr relationship with adverse cardiovascular events; (4) LASr was compared with traditional echocardiographic measures (when available); (5) at least one of the following outcomes was included in the analysis: major adverse cardiovascular events (MACE), death due to cardiovascular causes, all-cause death, AF, hospitalization for heart failure, exercise capacity, mean pulmonary arterial wedge pressure; (6) duration of follow-up was at least six months if outcomes investigated were all-cause death, CV death, and MACE.

Several critical exclusion criteria were set: studies published in languages other than English; letters, editorials, case reports, meta-analyses, or the inability to extract data.

### 2.3. Data Extraction

The following data were extracted from included studies: year of publication, study design, number of patients, patients’ age, clinical setting, CKD definition, outcomes investigated, duration of follow-up, odds ratio (OR), risk ratio (RR), hazard ratio (HR), confidence intervals (CIs), *p*-value, predictive power, and AUC—when available. Whenever possible, data are presented as percentages, mean or median values, ranges of variation. Disagreements were resolved by discussion and consensus.

### 2.4. Quality Assessment

We assessed the quality of non-randomized studies in the systematic review using the Newcastle-Ottawa scale, a star-based tool that evaluates studies at three different levels: selecting groups, comparability of groups, and outcome of interest [32]. It comprises eight essential items, for which stars are assigned, and the quality is judged according to the total number of stars. In studies without a control group, quality was assessed using a tool designed by the National Institutes of Health (NIH), encompassing 14 key questions [33].

## 3. Results

**Figure 1:**
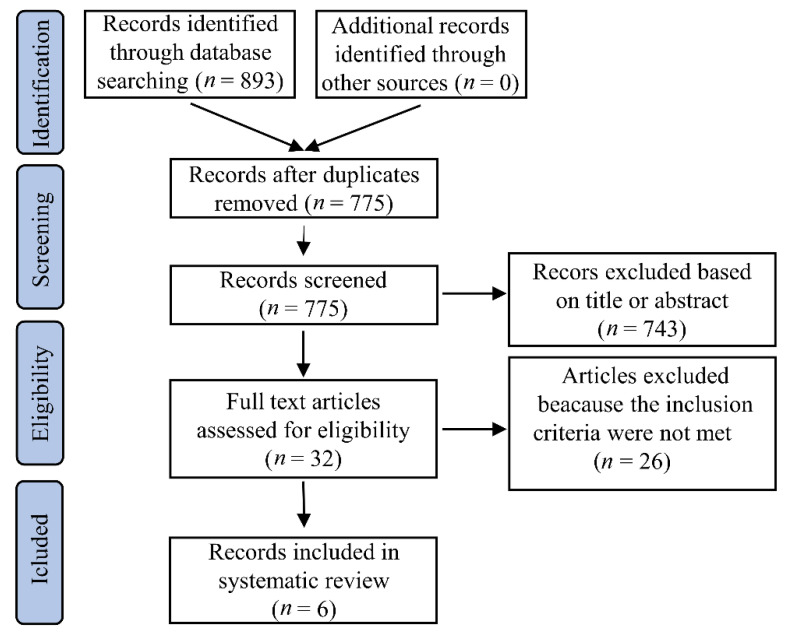
Flow diagram of selected studies for inclusion in the systematic review.

We searched the prespecified databases and identified 893 references. After screening for duplicates, 118 citations were excluded. Additional 743 citations were excluded based on title and abstract, leaving 32 articles for eligibility assessment. Six studies were included in our systematic review after excluding 26 references because the inclusion criteria were not met (Figure 1).

The characteristics of studies and population included and the outcomes measured were summarized in Table 1.

Of the included studies, three were performed in Australia [34,35,36], one—in Greece [37], one—in China [38], and one—in Turkey [39]. Additionally, two studies were performed in two tertiary hospitals [34,35]. The outcomes investigated were different across studies: MACE, all-cause death or CV death [34,36], exercise capacity [35], paroxysmal AF [37], diastolic dysfunction [38], and estimated pulmonary capillary wedge pressure [39]. Moreover, three studies included patients with ESKD only [37,38,39], while the rest included patients with CKD in different stages, mainly stages 3 and 4. Results reported in studies included in the systematic review were summarized in Table 2.

Gan et al. [34] evaluated LASr as a predictor for MACE (AF, heart failure, myocardial infarction, coronary revascularization and non-fatal stroke), CV mortality and all-cause mortality in patients with CKD stage 3–4, without pre-existing cardiac disease. During the follow-up interval, 54 adverse events were documented (deaths, *n* = 8; MACE, *n* = 46). Of all echocardiographic parameters investigated, only LASr was associated with the primary outcome (after adjustment for other variables, HR 0.89, 95% CI, 0.84–0.93, *p* < 0.01). Regarding the prediction of the primary outcome, LASr had a better discriminatory power, with AUC 0.84 (95% CI, 0.7610.900, *p* < 0.001), in comparison with LV global longitudinal strain (LVGLS)—AUC 0.696 (95% CI, 0.605–0.776, *p* = 0.016), LA volume index (LAVI)—AUC 0.671 (95% CI, 0.580–0.754, *p* < 0.001) and LV mass index (LVMI)—AUC 0.658 (95% CI, 0.566–0.741, *p* = 0.006). Moreover, patients with LASr ≤ 20.46 were at particular high risk, as 42% of them had an adverse event over three years (MACE or CV death).

Gan et al. [35] explored the utility of echocardiographic parameters (including LASr) in evaluating CKD patients’ exercise capacity without pre-existing cardiac disease. Although three parameters (LASr, E/e’ and LAVI) were initially associated with metabolic equivalents (METs) achieved, after integrating them in the final clinical model, only LASr remained an independent predictor of METs achieved (*p* < 0.01). The predictive performance of LASr was better (AUC 0.83, 95% CI, 0.78–0.88) than in case of E/e’ ratio during exercise (AUC 0.79, 95% CI, 0.73–0.85) or rest (AUC 0.67, 95% CI, 0.60–0.73), suggesting a clear advantage of LASr.

Kadappu et al. [36] evaluated the correlation between LASr and MACE, reporting similar results: LAGS being associated with an increased risk of MACE (*p* = 0.006). Notably, this study included stage 3 CKD patients, highlighting the usefulness of LASr even in the early stages of renal disease. Other parameters associated with MACE during follow-up were LAVI (*p* = 0.04) and LV late diastolic strain rate (LVSRA) (*p* = 0.03).

The relationship between LASr and the risk of paroxysmal AF in patients with ESKD and hemodialysis was explored by Papadopoulos et al. [37]. The authors included patients with preserved LV systolic function and excluded those with cardiac structural abnormalities (altering LA anatomy). After univariate analysis, several echocardiographic measures were found to increase the risk of AF: LASr (*p* = 0.003), LASr rate (*p* = 0.001), E/e’ (0.005), LAVI (*p* = 0.05) and LVMI (*p* = 0.005). In the multivariate analysis, the LASr rate remained strongly associated with an increased AF risk (*p* = 0.010). Nevertheless, limited by a small number of patients (*n* = 79), LASr proves to be a better marker than traditional echocardiographic measures in the CV risk stratification of ESKD patients.

A case-control study dealing with ESKD patients and preserved ejection fraction without CV disease symptoms investigated the relationship between peak LA longitudinal strain (PALS) and diastolic dysfunction [38]. Interestingly, PALS was reduced in ESKD compared to controls, even when LA pressure was normal (40.23 ± 12.72, *p* < 0.05). Additionally, PALS was significantly reduced in patients with diastolic dysfunction grade I (36.37 ± 8.59, *p* < 0.05) and grade II (33.33 ± 9.30, *p* < 0.05). Moreover, eGFR was independently correlated with PALS (*B* = 0.084, *p* = 0.046).

Altekin et al. [39] evaluated the association between LASr and pulmonary capillary wedge pressure (PCWP) based on echocardiographic measures. Notably, the authors investigated LASr parameters according to the cardiac cycle timing (LA_S-S_, left atrial systolic strain; LA_S-A_, left atrial late diastolic strain; LA_S-E_, left atrial early diastolic strain). All three components of LASr were strongly associated with PCWP, suggesting that LASr could be used as a marker to predict LV dysfunction in ESKD patients. However, this study limitation is represented by estimating PCWP using a formula, which could differ from the values measured invasively.

The quality assessment using the Newcastle–Ottawa scale and NIH tool for observational studies was presented in Tables S1 and S2. Overall, as none of the studies was randomized, the quality was judged as fair to low.

## 4. Discussion

To the best of our knowledge, this systematic review is the first one that included studies exploring the relationship between LASr and cardiovascular outcomes in patients with CKD.

LA structure and function evaluation gained interest in the last years, as it could be an early marker of a complex underlying cardiac disease with prognostic implications. LA modulates the LV preload by acting as a reservoir, conduit, and booster pump directly related to LV compliance and function. In the last years, LA function was extensively studied for its potential in risk stratification of patients with/without CVD with severe clinical implications [40].

The Dallas Heart Study [41] showed that the maximum LA volume and LA emptying fraction were associated independently with all-cause mortality in various clinical models in the general population during eight years of follow-up. However, LA parameters were evaluated by cardiac magnetic resonance, a more expensive and less available technique than echocardiography. In another study involving the general population [42], LASr was associated with a composite cardiovascular outcome (ischemic heart disease, heart failure, or CV death) but only in women (HR 1.46, 95% CI, 1.05–2.02, *p* = 0.025).

Another relevant clinical implication of LASr in the general population is represented by AF prediction [43]. Individuals with lower PALS were at a higher risk of AF at univariable analysis, effect maintained significant only in people aged < 65 years (after multivariable analysis, HR 1.46, 95% CI, 1.06–2.02, *p* = 0.021, for each 5% PALS decrease). Moreover, LASr was linked to an increase in thrombotic events and unsuccessful electrical cardioversion in patients with AF or atrial flutter, suggesting the utility of LASr in therapeutic decision-making regarding rhythm control [44]. LA deformation parameters could guide anticoagulant therapy initiation, as LASr measures were associated with increased stroke risk [45].

Despite all promising clinical applications of LA parameters mentioned above, few studies validated LASr role in CKD. Two studies included in our systematic review reported reliable correlations between LASr and MACE or (all-cause) mortality.

One of them evaluated the link between LASr and long-term CV outcomes (3.9 ± 2.7 years) in patients with CKD stage 3–4 [34]. Although patients with prior cardiac disease were excluded, the prevalence of CV risk factors was high both in patients without CV events and those with CV events: hypertension (93% vs. 96%), hypercholesterolemia (75% vs. 76%), diabetes mellitus (46% vs. 63%), obesity (29% vs. 37%), anemia (27% vs. 35%), thus representing a high-risk cohort of patients. Given all echocardiographic parameters, only LASr remained associated with the primary outcome (CV death and MACE) after multivariable analysis. LVMI, LAVI, and even LVGLS did not reach statistical significance.

The other study evaluating LASr and MACE correlations showed similar results, as LAGS was associated with an increased risk of MACE [36]. Nevertheless, the MACE definition was different from the previous study, as it included death, CV events, or ESKD. Additionally, only patients with stage 3 CKD were included.

These data support the utility and feasibility of LASr parameters in clinical practice for an accurate CVD risk stratification of CKD patients (even in the early stages). Furthermore, LASr could be integrated into future prediction models and other clinical and imagistic variables, as it could detect CKD patients at high risk even better than LVGLS.

A “softer” CV outcome associated with LASr is exercise capacity. Only LASr was significantly associated with exercise capacity among echocardiographic measures in a final prediction model, with an excellent predictive power for achieved METs [35]. Moreover, age, female gender, and body mass index were other clinical variables associated with a reduced exercise capacity. So, LASr parameters might be regarded as potential markers to predict the occurrence and evolution of CKD patients’ symptoms.

Moreover, LA deformation imaging was associated with LV diastolic dysfunction even in patients with ESKD and preserved LV ejection fraction [38]. A gradual reduction of PALS was noticed along with diastolic dysfunction progression (*p* < 0.001). Conversely, LAVI and LVMI were higher in the presence of diastolic dysfunction (*p* < 0.05).

Finally, an attractive application of LA deformation imaging is represented by LV filling pressures estimation. Patients with ESKD, hemodialysis, and preserved LV ejection fraction, showed a strong correlation between LASr and estimated PCWP [39]. This study particularity is represented by the fact that the authors investigated all three components of LASr, in concordance with cardiac cycle time intervals (LA_S-S_, LA_S-E_, LA_S-A_); each of them being associated with estimated PCWP.

The main limitations of the LASr technique are represented by echocardiographic image quality, inter-observer variability, the necessity of software, and the learning curve. Probably, the necessity of dedicated software makes LA deformation imaging less available nowadays. Moreover, a different software application could be a source of variability [46]. Data regarding the learning curve can be extrapolated from LV strain imaging since a minimum of 50 examinations are required for becoming proficient in LVGLS assessment [47].

## 5. Conclusions

In the era of personalized medicine and novel imagistic techniques, LASr constitutes a promising marker for CV events prediction and risk evaluation in CKD settings. Critical clinical applications of LASr in CKD include detecting patients at high risk of MACE, CV mortality, and all-cause mortality to individualize the follow-up strategy or therapeutic regimen. AF prediction using LASr holds critical practical implications so that anticoagulant therapy could be initiated promptly. The inclusion of LASr in the future clinical models might improve the prediction power; however, more prospective studies are needed.

## Figures and Tables

**Table 1:** General characteristics of studies included in present systematic review.

Author, Year	Design	Patients, No	Age (years), Median/Mean	Parameters	Software	Setting	Outcomes	Follow-Up
Gan et al., 2020	Observational, prospective	243	59.2 ± 14.4	LASr E/e’ LV mass LAV LVGLS	EchoPAC Version BT13, GE	CKD stage 3–4, without previous cardiac disease	Primary end point: CV death and MACE Secondary end point: composite of all-cause death and MACE	3.9 ± 2.7 years
Gan et al., 2021	Observational, prospective	218	63.9 ± 11.7	LASr LVGLS LV mass LAVI E/A E/e’	EchoPAC Version BT13, GE Healthcare	CKD stage 3–4, without prior cardiac history	Exercise capacity	NA
Papadopoulos et al., 2018	Observational, prospective	79	57 ± 17	LASr LASr rate LAVI LVMI E/e’	EchoPAC Version 113, GE Healthcare	ESRD + HD, preserved LV systolic function	Paroxysmal atrial fibrillation	16 ± 5 months
Kadappu et al., 2016	Observational, prospective	86	–	LAGS LVSRa LAVI	NA	CKD stage 3	Composite of MACE (death, CV events or ESRD)	60 months
Li et al., 2019	Observational	59 ESRD	44.41 ± 16.28 ESRD—group C	PALS LA stiffness LAVI LVMI	Philip Qlab 10.0, Andover	ESRD, LVEF > 50%, without symptoms of CV disease	Diastolic dysfunction	NA
		30 healthy controls	40.55 ± 11.4 controls					
Altekin et al., 2013	Observational	85 ESRD	33.79 ± 9.08 ESRD	LASr LA stiffness LAVI LVMI E/A E/e’ E-DecT IVRT	EchoPAC Version 8, GE Healthcare	ESRD + HD, preserved LVEF	Estimated pulmonary capillary wedge pressure	NA
		60 healthy controls	39.17 ± 10.08 healthy controls					

CKD—chronic kidney disease; CV—cardiovascular; E-DecT—E wave deceleration time; ESRD—end-stage renal disease; HD—hemodialysis; IVRT—isovolumetric relaxation time; MACE—major adverse cardiovascular events; LA—left atrium; LAGS—left atrial global strain; LASr—left atrial strain; LAV—left atrial volume; LAVI—left atrial volume index; LV—left ventricle; LVEF—left ventricular ejection fraction; LVGLS—left ventricular global longitudinal strain; LVMI—left ventricular mass index; LVSRa—left ventricular late diastolic strain rate; PALS—peak left atrial longitudinal strain.

**Table 2:** Results reported in studies included in present systematic review.

Study, Year	Outcomes	Parameter	Results	
Gan et al., 2021	Cardiovascular death and MACE	LASr	HR 0.89 (95% CI, 0.840.93)	*p* < 0.01
		LAVI	HR 1.02 (95% CI, 0.99–1.05)	*p* = 0.31
		E/e’	HR 1.03 (95% CI, 0.98–1.09)	*p* = 0.25
	Composite of all-cause death and MACE	LASr	HR 0.87 (95% CI, 0.82–0.92)	*p* < 0.01
		LAVI	HR 1.01 (95% CI, 0.98–1.04)	*p* = 0.44
		E/e’	HR 1.04 (95% CI, 0.98–1.11)	*p* = 0.22
Gan et al., 2021	Reduced exercise capacity	LASr	*r* = 0.70	*p* < 0.01
			AUC, 0.83 (95% CI, 0.78–0.88)	*p* < 0.01
		E/e’ (exercise)	*r* = −0.65	*p* < 0.01
			AUC, 0.79 (95% CI, 0.73–0.85)	*p* < 0.01
		E/e’ (resting)	AUC, 0.67 (95% CI, 0.60–0.73)	*p* < 0.01
		LAVI	*r* = −0.18	*p* < 0.01
Papadopoulos et al., 2018	Paroxysmal atrial fibrillation	Mean LASr	OR 0.847 (95% CI, 0.760–0.944)—univariate analysis	*p* = 0.003
		Mean LASr rate	OR 0.03 (95% CI, 0.002–0.416)—multivariate analysis	*p* = 0.010
		LAVI	OR 1.049 (95% CI, 1.000–1.101)	*p* = 0.05
Kadappu et al., 2016	MACE (death, cardiovascular events, ESRD)	LAGS	HR 3.8 (95% CI, 1.5–9.9)	*p* = 0.006
		LVSRa	HR 2.9 (95% CI, 1.1–7.5)	*p* = 0.03
		LAVI	HR 0.38 (95% CI, 0.16–0.94)	*p* = 0.04
Li et al., 2019	Diastolic dysfunction	PALS	33.33 ± 9.30 (grade II diastolic disfunction) vs. 51.75 ± 5.82 (control group)	*p* < 0.05
			36.37 ± 8.59 (grade I diastolic dysfunction) vs. 51.75 ± 5.82 (control group)	*p* < 0.05
Altekin et al., 2013	Estimated pulmonary capillary wedge pressure	LA_S-S_	β 0.409 (95% CI, 0.140–0.246)	*p* < 0.001
		LA_S-E_	β −0.125 (95% CI, −0.139–−0.019)	*p* = 0.01
		LA_S-A_	β 0.461 (95% CI, 0.3–0.498)	*p* < 0.001

AUC—area under the curve; ESRD—end-stage renal disease; LA_S-A_—left atrial late diastolic strain; LA_S-E_—left atrial early diastolic strain; LA_S-S_—left atrial systolic strain; LAGS—left atrial global strain; LAVI—left atrial volume index; LASr—left atrial strain; LVSRa—left ventricular late diastolic strain rate; MACE—major adverse cardiovascular events; PALS—peak left atrial longitudinal strain.

## Supplementary Materials

The following are available online at https://www.mdpi.com/article/10.3390/diagnostics11040671/s1, Table S1. Quality assessment using NIH tool for observational studies, Table S2. Quality assessment using Newcastle-Ottawa scale for non-randomized studies.
