# Supplementary material for: A Systematic Review on the Correlations between Left Atrial Strain and Cardiovascular Outcomes in Chronic Kidney Disease Patients
Source: Diagnostics (Basel). 2021 Apr 8;11(4):671. doi: 10.3390/diagnostics11040671 (PMC8068338; doi:10.3390/diagnostics11040671)
Supplement: Supplementary file 1 [file diagnostics-11-00671-s001.pdf]

Table S1. Quality assessment using NIH tool for observational studies.

Author, year	1	2	3	4	5	6	7	8	9	10	11	12	13	14	Quality
Gan et al, 2021 [34]	Y	Y	Y	Y	NR	Y	Y	NA	NR	N	Y	NA	NR	Y	Fair
Gan et al, 2021 [35]	Y	Y	Y	Y	NR	Y	Y	NA	NR	N	Y	NA	NR	Y	Fair
Papadopoulos et al, 2018 [37]	Y	Y	Y	Y	NR	Y	Y	NA	NR	N	N	NA	NR	Y	Fair
Kadappu et al, 2016 [36]	Y	Y	NR	Y	N	Y	Y	NA	Y	N	Y	NA	NR	N	Poor

Y – yes; N – no; NA – not applicable; NR – not reported. **1** – Was the research question or objective in this paper clearly stated? **2** – Was the study population clearly specified and defined? **3** – Was the participation rate of eligible persons at least 50%? **4** – Were all the subjects selected or recruited from the same or similar populations (including the same time period)? Were inclusion and exclusion criteria for being in the study prespecified and applied uniformly to all participants? **5** – Was a sample size justification, power description, or variance and effect estimates provided? **6** – For the analyses in this paper, were the exposure(s) of interest measured prior to the outcome(s) being measured? **7** – Was the timeframe sufficient so that one could reasonably expect to see an association between exposure and outcome if it existed? **8** – For exposures that can vary in amount or level, did the study examine different levels of the exposure as related to the outcome (e.g., categories of exposure, or exposure measured as continuous variable)? **9** – Were the exposure measures (independent variables) clearly defined, valid, reliable, and implemented consistently across all study participants? **10** – Was the exposure(s) assessed more than once over time? **11** – Were the outcome measures (dependent variables) clearly defined, valid, reliable, and implemented consistently across all study participants? **12** – Were the outcome assessors blinded to the exposure status of participants? **13** – Was loss to follow-up after baseline 20% or less? **14** – Were key potential confounding variables measured and adjusted statistically for their impact on the relationship between exposure(s) and outcome(s)?

Table S2. Quality assessment using Newcastle-Ottawa scale for non-randomized studies.

	Li et al, 2019 [38]	Altekin et al, 2013 [39]
Selection		
Is the case definition adequate?	*	*
Representativeness of the cases	*	*
Selection of controls		
Definition of controls	*	*
Comparability		
Comparability of cases and controls on the basis of the design or analysis	*	*
Exposure		
Ascertainment of exposure	*	*
Same methods of ascertainment for cases and controls	*	*
Non-response rate		
Total	6	6

Good quality: 3 or 4 stars in selection domain AND 1 or 2 stars in comparability domain AND 2 or 3 stars in outcome/exposure domain. Fair quality: 2 stars in selection domain AND 1 or 2 stars in comparability domain AND 2 or 3 stars in outcome/exposure domain. Poor quality: 0 or 1 star in selection domain OR 0 stars in comparability domain OR 0 or 1 stars in outcome/exposure domain.
